# Assessing health-related quality of life in young Japanese children with chronic conditions: Preliminary validation of the DISABKIDS smiley measure

**DOI:** 10.1186/s12887-017-0854-4

**Published:** 2017-04-05

**Authors:** Hatoko Sasaki, Naoko Kakee, Naho Morisaki, Rintaro Mori, Ulrike Ravens-Sieberer, Monika Bullinger

**Affiliations:** 1 Department of Health Policy, National Center for Child Health and Development, Tokyo, Japan; 2 Division of Bioethics, National Center for Child Health and Development, Tokyo, Japan; 3 Department of Social Medicine, National Center for Child Health and Development, Tokyo, Japan; 4 Department of Child and Adolescent Psychiatry, Psychotherapy, and Psychosomatics, University Medical Center Hamburg-Eppendorf, Hamburg, Germany; 5 Department of Medical Psychology, University Medical Center Hamburg-Eppendorf, Hamburg, Germany

**Keywords:** Young children, Chronic conditions, Health-related quality of life, Assessment, Psychometric testing

## Abstract

**Background:**

Although there is an increasing need to investigate the health-related quality of life (HRQOL) of children and adolescents with chronic conditions in Japan, there is currently no standardized measure in which young children can directly answer questions about their HRQOL. The DISABKIDS Smiley measure uses face emoticons to measure HRQOL and distress caused by illness and related treatments among young children. We tested the reliability and validity of the DISABKIDS Smiley measure in a sample of young Japanese children.

**Methods:**

After translating the child and parent questionnaires into Japanese, a pre-test was performed to test the content validity in accordance with guidelines from the DISABKIDS Group. In total, 60 child-parent pairs were recruited to participate in the survey. We measured internal consistency of the scales using Cronbach’s alpha as well as Guttman split-half, test-retest reliability using intraclass correlation coefficients (ICCs) at a two-week interval, and ICCs between child- and parent-reported scores. Convergent validity of the scale was also examined against the Kiddy-KINDL scale.

**Results:**

Both child-reported and parent-reported scales showed good internal consistency and split-half reliability. Test-retest reliability of the child-reported version (ICC = 0.53, *p* = 0.004) was lower than that of the parent-reported version (ICC = 0.80, *p* < 0.001). Moderate to good agreement between child- and parent- reported scales was observed in both the first (ICC = 0.75, *p* < 0.001) and second administration (ICC = 0.71, *p* < 0.001). Moderate to very strong positive correlations were observed with the total score of the Kiddy-KINDL child-reported version (*r* = 0.51, *p* < 0.001), and facets of the Kiddy-KINDL parent-reported version (ranging from *r* = 0.364 to *r* = 0.60, *p* < 0.001) and total score (*r* = 0.71, *p* < 0.001).

**Conclusions:**

The psychometric property of the instrument showed that the Japanese version of the DISABKIDS Smiley can be applied to assess the HRQOL of Japanese children with chronic conditions. Further investigation will be needed to explore the reliability and validity for repeated use of the instrument in clinical practice as an indicator of clinical significance.

## Background

Chronic conditions have been shown to have a negative impact on psychosocial health, academic functioning and social competence in children [1, 2]. Children treated for chronic conditions often experience lengthy stays in hospital, medical treatment that can be uncomfortable and painful, disrupted school attendance, and limits on social and physical activities [3], which may lower their psychosocial well-being. With increasing consideration of patient perspectives for better care, there is a need to address patient-reported outcomes (PROs), particularly on health-related quality of life (HRQOL). HRQOL is generally considered to be a multidimensional construct covering physical, emotional, mental, social, and behavioural components of well-being and function as perceived by patients or proxies [4]. One of the objectives to assess PROs is to take into account healthcare needs that are not typically identified by medical indicators, and more importantly, to help patients manage their chronic conditions over the course of long-term treatment.

In many cases, parental reports are needed to complement child-reported HRQOL, especially for younger children whose language skills and time perceptions are still developing. However, parents and children do not necessarily share the same evaluations of HRQOL, particularly regarding non-observable inner states (e.g. emotional or social HRQOL) [5]. The agreement between parent and child responses may differ by sample populations and measurements [6]. Therefore, it is important not only to obtain information from the parent but also from the child’s perspective. Although young children’s understanding of the concept of HRQOL is not yet properly developed, children are able to identify states of wellbeing and illness, can respond to questions read aloud to them, and can report on their feelings [7]. User-friendly surveys for children are facilitated by simple and clear questions, age-appropriate vocabulary, and fewer response items so children can answer questions easily [8].

Despite the importance of better understanding the psychosocial impact of chronic illness and its associated medical treatment on Japanese children’s HRQOL, there is currently no standardized measure in Japan that specifically gauges young children’s own perspectives. A systematic review recently conducted by the National Institute for Health Research in the United Kingdom [9] concluded that out of 25 patient-reported outcome measures for children and young people, only the DISABKIDS Smiley was found to show reasonable evidence of psychometric performance. The DISABKIDS Smiley measure [4, 7] was developed as part of the DISABKIDS European Project to provide a cross-culturally valid assessment for the HRQOL of children aged 4 to 7 years with chronic conditions. The instrument has been validated in eight languages to assess HRQOL using both a child report and a parent-observer report [10]. The DISABKIDS Smiley measure enables young children to directly answer questions by selecting ‘smileys’ or face emoticons to rate their HRQOL regarding the possible consequences of chronic conditions, while a generic HRQOL instrument cannot capture the aspects. This measure might be preferable to a generic HRQOL instrument with multi domains such as the Kiddy-KINDL [11] because it is simple and fast to complete for children with chronic conditions in a developmental stage. This study aims to test the reliability and validity of the DISABKIDS Smiley measure in a sample of Japanese children. We translated The DISABKIDS Smiley measure into Japanese, and evaluated its internal consistency and convergent validity.

## Methods

### Participants

Children aged 4 to 7 years were eligible for inclusion if they: (1) had been receiving treatment for a chronic condition for at least 6 months; (2) visited their doctor regularly, and (3) were willing to participate in the study. As the original European study of the DISABKIDS measure [4, 7] included participants with asthma and/or allergies, we included these conditions in this study because asthma and/or allergies are also common conditions among Japanese children. Children who were diagnosed with the chronic conditions of asthma and/or allergies, dermatitis, respiratory disease, or growth hormone deficiency and their parents were recruited from the outpatient clinic at the National Center for Child Health and Development, Tokyo, Japan. Children who had difficulty responding to questions due to intellectual disability, and those who did not give consent to participate, were excluded. The study aimed to include a balanced distribution of ages and sex.

### Ethical considerations

Participation in the pre-test cognitive interviews and field tests was voluntary. Verbal consent was obtained from children aged 4 to 7 years and a written consent form was completed by their parents. Anonymity and confidentiality of data was assured to all participants. The pre-test and field-test phases were approved by the Ethical Review Board of the National Center for Child Health and Development, Tokyo, Japan on 1 November 2013 and 27 December 2013, respectively.

### Procedure

We followed all the steps in the Translation & Validation Procedure provided by the European DISABKIDS Group [12]. The translation methodology included the following specific procedures: (1) production of two independent forward translations; (2) reconciliation of items into a single forward-translation version; (3) back-translation; (4) review of forward- and back-translations; (5) assessment of conceptual equivalence/first harmonization of problematic items; (6) semantic validation (cognitive interviews); and (7) international harmonization under the supervision of the European DISABKIDS Group coordination [12]. The guideline also recommends testing the final translation of the DISABKIDS instrument in a validation study in order to obtain more information about the psychometric properties of the instrument in the relevant language [12].

#### Translation and pre-test (cognitive interviews)

After successful review of the forward-back translation, a harmonization session was conducted with a member of the DISABKIDS Group to evaluate the final forward translation. Pre-testing of six children of both sexes and their parents was then conducted using the Japanese translation to test content validity. Participants were asked whether the items of the questionnaire were comprehensible and acceptable. This process was undertaken from October to December 2013.

#### Field test

In total, 60 children and one of their parents were recruited to participate in the survey. Research assistants explained the purpose and procedure of the study to all participants. If consent was obtained from both the child and their parent, the child was asked to answer the child-reported version of the questionnaire with assistance from his or her parent. The parent was asked to answer the parent-reported version while they waited to see a doctor. After completion of the questionnaire, the parents received retest questionnaires and returned them by mail 2 weeks after the first administration.

### Instrument

#### Socio-demographic information

We asked parents to fill out a form attached to the parent-reported version in order to obtain their socio-demographic information. The form contained questions on the following points: (1) who is responding to the questionnaire; (2) age of the respondent; (3) marital status of the respondent; (4) how much the respondent feels financially comfortable (economic status); (5) who is the main carer of the child; and (6) the child’s number of siblings. We chose to assess economic status using descriptive categories instead of ranges of income. As some people may hesitate to indicate their level of income, we excluded income from the response items to avoid a situation where parents may refuse to respond to the questionnaire.

#### DISABKIDS smiley measure

The DISABKIDS Smiley measure [4, 7] was developed to assess HRQOL among children with chronic disease. The measure is a six-item scale that covers a single HRQOL domain. Response items to questions are denoted with face emoticons that each represent a possible emotional state or level of agreement ranging from an extremely sad face emoticon, meaning ‘very unhappy’, to a smiling emoticon, meaning ‘very happy’. The child-reported and parent-reported versions contain the same items as follows: (1) “I feel...”/“My child feels …”; (2) “When I go to the doctor, I feel ...”/“When my child goes to the doctor, he/she feels …”); (3) “When I do things on my own, I feel ...”/“When my child does things on their own, they feel …”; (4) “I feel about myself…”/“About him/herself, my child feels ….”; (5) “Kindergarten or school makes me feel …”/ “Kindergarten or school makes my child feel ...”; (6) When I compare myself to other children I feel ...”/“When my child compares him/herself to other children, he/she feels …”). Parents were also asked about the clinical conditions of their children in the parent-reported version. The DISABKIDS Group reported a Cronbach’s alpha of 0.69 (retest = 0.70) for the child-reported version and 0.71 for the parent-reported version. Intraclass correlation coefficients (ICCs) between test and retest scores were 0.69 [4].

#### Kiddy-KINDL questionnaire

The generic Kiddy-KINDL Questionnaire [11, 13] was developed to measure HRQOL independent of health status in children aged 4 to 7 years using a child-reported questionnaire and a parent-observer version. The child-reported version is a 12-item scale that consists of 6 dimensions: physical well-being, emotional well-being, self-esteem, family, friends, and school, with a 3-point Likert scale ranging from 1 = “never” to 3 = “very often” to form a total score. The 46-item parent-reported version consists of 6 subscales with a 5-point Likert scale ranging from 1 = “never” to 5 = “all the time”. The scores of each facet and the total score were calculated for the parent-reported version. Nemoto et al. [13] translated and validated the measure for Japanese children, and reported a Cronbach’s alpha reliability of 0.70 for children’s responses and 0.88 for parents’ responses. Psychometric testing by Nemoto et al. [13] showed that the Japanese version of the Kiddy-KINDL Questionnaire is useful for assessing the HRQOL of young children in Japan [13]. The Kiddy-KINDL Questionnaire was administered to test convergent validity of the Japanese DISABKIDS Smiley measure.

### Statistical analysis

All statistical analyses were undertaken in SPSS version 21.0 (IBM Corporation, USA).

### Item analysis and reliability

The normality of response distribution for each item was checked using skewness, and then means, standard deviations (SDs), minimum-maximum values were calculated. Internal consistency of the scale was assessed with Cronbach’s alpha and the Guttman split-half reliability coefficient. Test-retest reliability was tested using ICCs at a two-week interval, and ICCs between child- and parent-reported versions were obtained for average measurements within a two-way mixed model (absolute agreement).

### Validity

Convergent validity of the scale was examined via correlations of the Kiddy-KINDL scores assessing similar concepts with aspects of the DISABKIDS Smiley. Pearson correlation coefficients were calculated between the scores of DISABKIDS child-reported questionnaires and the total score of the Kiddy-KINDL child-reported questionnaire, the score of the DISABKIDS parent-reported version and the facet and total scores of the Kiddy-KINDL parent-reported version.

## Results

### Translation and pre-test (cognitive interviews)

The translation process was successfully completed in accordance with relevant guidelines [11]. Children and parents made comments and suggestions during the cognitive interviews. For example, it was difficult for children to understand the difference between “I feel…” and “I feel about myself …..”. To clarify this difference, “I feel…” was changed to “Now, I feel…”. In the list of chronic conditions, “dermatitis” was supplemented by “atopic dermatitis” in parentheses. There were no indications of a need to omit items from the scales, or to add new concepts of HRQOL, and except for the changes noted above, no additional changes were made to the scales.

### Field test

#### Description of sample

Table 1 shows the descriptive statistics of the study sample including frequencies of socio-demographic and clinical variables. A total of 60 children with chronic conditions and 60 parents/caregivers completed the test and retest versions. The ages of children ranged from 4 to 7 years with a balanced distribution of child age as follows: 4 years (26.7%), 5 years (25.0%), 6 years (26.7%), and 7 years (21.7%). The mean age of parents was 37.9 years.

**Table 1:** Socio-demographic and clinical characteristics of the sample

Parent Characteristics	(n/%)
Respondent	
Mother	51 (86.4)
Father	8 (13.6)
Age (mean/SD)	37.8 (4.3)
Marital status: married	53 (91.2)
Economic status (living condition)	
Very comfortable	2 (3.6)
Somewhat comfortable	30 (53.6)
Somewhat difficult	20 (35.7)
Very difficult	4 (7.1)
Child Characteristics	
Age (mean/SD)	5.5 (1.1)
Sex	
Male	38 (63.3)
Female	22 (36.7)
Number of siblings^a^	
More than one	1
One	41
None	21
Clinical condition	
Asthma and/or allergies	50 (83.3)
Circulatory disease	7 (11.7)
Kidney disease	2 (3.3)
Growth hormone deficiency	1 (1.7)

^a^multiple answers allowed

#### Item analysis and reliability

Distributional characteristic of scale scores for child- and parent-reported versions are given in Table 2. The internal consistency reliabilities ranged from α = 0.66 (child-reported version) to α = 0.84 (parent-reported version). The Guttman split-half reliability reached a coefficient of 0.63 for the child-reported version and 0.81 for the parent-reported version. The test-retest reliability coefficients were 0.53 (*p* = 0.004) for the child-reported version and 0.80 (*p* < 0.001) for the parent-reported version. Table 3 shows that ICCs were significant between child-reported scores and parent-reported scores for both the first administration (ICC = 0.75, *p* < 0.001) and the second administration (ICC = 0.71, *p* < 0.001).

**Table 2:** Psychometric properties of the total score of the Japanese DISABKIDS-Smiley measure for child- and parent-reported versions

DISABKIDS-Smiley	N	Min.	Max.	Mean	SD	Skewness	α	Split-half	ICC*
Child-reported version	60	45.8	100.0	75.2	14.7	−.06	.66	.63	.53 (*p* = 0.004)
Child-reported version (retest)	50	41.7	100.0	72.3	15.2	−.11	.63	.60	
Parent-reported version	60	33.3	100.0	71.3	16.0	−.13	.84	.81	.80 (*p* < 0.001)
Parent-reported version (retest)	50	20.8	100.0	70.5	14.6	−.65	.85	.88	

ICC*: the test-retest intraclass correlation coefficients

**Table 3:** Intraclass correlation coefficients (ICC) between child-reported version and parent-reported version of the Japanese DISABKIDS-Smiley measure

	ICC	P
Test	0.75	*p* < 0.001
Retest	0.71	*p* < 0.001

#### Validity

Table 4 shows the results of the convergent validity analyses. A significant and positive correlation was found between the total score of the DISABKIDS Smiley child-reported version and the total score of the child-reported Kiddy-KINDL (*r* = 0.51, *p* < 0.001). Statistically significant associations were also observed between the total score of the DISABKIDS Smiley parent-reported version and the parent-reported Kiddy-KINDL facets (ranging from *r* = 0.36 to *r* = 0.60, *p* < 0.001) and total score (*r* = 0.71, *p* < 0.001).

**Table 4:** Correlation coefficients (Pearson r) for total score of the child-reported version and parent-reported version of DISABKIDS compared with the Kiddy-KINDL

	Kiddy-KINDL						
DISABKIDS	Physical well-being	Emotional well-being	Self-esteem	Family	Friends	School	Total
Child-reported version	-	-	-	-	-	-	.51^**^
Parent-reported version	.45^**^	.60^**^	.59^**^	.36^**^	.60**	.58**	.71^**^

*P < 0.05 **P < 0.01

## Discussion

In this study, we examined the effectiveness of the DISABKIDS Smiley measure to assess HRQOL in Japanese children with chronic conditions. Our results revealed acceptable to good internal consistency and split-half reliability, moderate to good test-retest reliability of the DISABKIDS Smiley, and good external validity against the Kiddy-KINDL child-reported and parent-reported versions. Child-reported scores and parent-reported scores also showed moderate to good agreement.

The internal consistency of the child-reported version (α = 0.66) was lower than that of the parent-reported version (α = 0.84). The most likely problem relating to lower reliability of the scale is a reduction in statistical power. The test-retest reliability of the child-reported version was statistically significant (ICC = 0.53, *p* = 0.004) but lower than that of the parent-reported version (ICC = 0.80, *p* < 0.001). This may be due to the young age of the child participants, which is characterized by rapid cognitive and physical development, and limited perceptions of concepts such as time and language. This study showed moderate data agreement between the child-reported and the parent-reported versions of the DISABKIDS measure, which was not tested in the original European study [4]. Moderate to good agreement [14] was observed in both administrations of the measure (1st test administration ICC = 0.75, *p* < 0.001; 2nd retest administration ICC = 0.71, *p* < 0.001), which is a higher level of agreement than the Portuguese DISABKIDS-12 Chronic Generic Module (ICC = 0.54, *p* < 0.001) [15]. The combination of child and parent versions is strongly recommended to better assess and understand HRQOL in younger children.

Convergent validity was confirmed by assessing the correlations with Kiddy-KINDL. The correlation coefficients with the Kiddy-KINDL parent-reported version in a Japanese sample were higher than those of the original European study [4]. The results showed that the parent-reported version had better reliability and validity than the child-reported version.

This study has several limitations. First, the clinical conditions in the sample were mainly asthma and/or allergies. Second, this study was conducted with patients from only one Japanese facility. A sample of different clinical conditions from a variety of facilities may have yielded different psychometric properties. Third, the sample size is small and parental help in presenting the questions may also have influenced children’s responses. Finally, approaches to assess child-reported HRQOL in this age group are hampered by the early developmental stage of the child participants, and by significant differences in children’s verbal competence and reflective ability [7].

Concerning the application of the DISABKIDS Smiley measure in research and practice, the combined use of child- and parent-reported versions of the questionnaire may be advisable. Rather than expecting a single indicator of child HRQOL, this strategy acknowledges potential biases in each approach and makes it possible to represent both perspectives from children and parents. There is little research on HRQOL in children below the age of 8 years, especially regarding child-reported questionnaires [16]. The Kiddy-KINDL for children in the general population and the DISABKIDS Smiley for children with chronic conditions now provide two instruments ready for use in studies on the HRQOL of young children. Further investigation is needed to understand the descriptive, evaluative and predictive potential of the DISABKIDS Smiley measure and to identify cut-off values for impairments in HRQOL [7].

## Conclusion

Examination of the psychometric properties of the DISABKIDS Smiley measure indicates that the Japanese version is an adequate instrument to assess the HRQOL of young Japanese children with chronic conditions. Further investigation using large and diverse samples is needed to strengthen the use of the instrument in interventions. The instrument may be used in research and practice to better understand the wellbeing of chronically ill young children, to identify their needs for care, to examine the effects of therapeutic treatment on HRQOL, and to critically examine the quality of healthcare services. Assessing HRQOL also has the potential to improve patient-clinician communication and to obtain child and parent perspectives on HRQOL, which corresponds to the family orientation of clinical practice in pediatrics.

## Abbreviations

HRQOL: Health-related quality of life
ICCs: Intraclass correlation coefficients
PROs: Patient-reported outcomes
SDs: Standard deviations
SPSS: Statistical package for social sciences
